# Natural History, Pathology, and Treatment of the Epidemic Fever at Present Prevailing in Edinburgh and Other Towns

**Published:** 1844-07

**Authors:** 


ART. XV.

1. *Natural History, Pathology, and Treatment of the Epidemic Fever at present prevailing in Edinburgh and other Towns.* By JOHN ROSE CORMACK, M.D. F.R.S.E.—*Edinburgh*, 1843. 8vo, pp. 182.
2. *Remarks on the present Epidemic Fever.* By WILLIAM PULTENEY ALISON, M.D. F.R.S.E., &c. (*Scottish and North of England Medical Gazette*, vol. i, pp. 1 to 4.)—*Edinburgh*, 1843.
3. *Letter on the present Epidemic of Dundee.* By J. ARROTT, M.D. (*Ibid.* p. 129.)
4. *Clinical Observations on Fever.* By Professor HENDERSON. (*Ibid.* p. 162.)
5. *On the Presence of Urea in the Blood in the prevailing Epidemic Fever and in Typhus.* By MICHAEL W. TAYLOR, Esq. (*Ibid.* p. 289 to 293.) 1844.
6. *Remarks on the Epidemic Fever in Aberdeen during the year* 1843. By A. KILGOUR, M.D. (*Ibid.* p. 321.)
7. *On some of the Characters which distinguish the Fever, at present Epidemic, from Typhus Fever.* By Prof. HENDERSON. (*Edinburgh Medical and Surgical Journal*, vol. lix.)—*Edinburgh*, 1843.
8. *Some Account of the Epidemic Fever prevailing in Glasgow.* By DAVID SMITH, M.D. (*Ibid.* vol. lx, p. 67.)
9. *Notice of a Febrile Disorder which has prevailed at Edinburgh during the summer of* 1843. By DAVID CRAIGIE, M.D. (*Ibid.* vol. lx, p. 411.)
10. *Some Account of the Epidemic Remittent Fever prevalent in Glasgow in* 1843. By WILLIAM MACKENZIE, M.D. (*London Medical Gazette, Nov.* 24, 1843.)

No one can have devoted any degree of attention to the study of continued fevers, without arriving at a conviction that the principles upon which the different varieties have been grouped or classified are vague, vacillating, and contradictory. Without referring to the numerous subdivisions of different writers, the distinctions between the three species of Dr. Cullen's classification, generally followed in this country, *synocha*, *synochus*, and *typhus*, are so indefinite and ill understood, if in themselves accurate, that even our most precise systematic writers are to be found describing different degrees of the same fever, as illustrative of all the three varieties; while, with authors less precise, all the difficulties and distinctions have been evaded by describing all the varieties of fever under the common designation of typhus.

The identity of the typhus fever of this country and the typhoid of M. Louis and other continental writers was very fully discussed by us in a recent volume, (Vol. XII, p. 293-326,) and we then arrived at the conclusion, that the continued fevers of both countries were the *same species* of disease, although *different varieties* of that species; and that the French variety was occasionally, though rarely, observed in this country, and the British variety in France.

The identity of these two forms of fever has again been discussed at some length by Professor Bartlett, whose work was reviewed by us in our last Number. Dr. Bartlett recognizes only four species of fevers: "to wit, typhoid fever; typhus fever; periodical fever, in its three forms, of intermittent, bilious remittent, and congestive; and yellow fever." He recognizes no inflammatory fever, or any other form of continued fever, distinct from typhoid and typhus.

Without entering again into a subject fully examined by us in the articles referred to, we may remark briefly that there is nothing in Dr. Bartlett's book which would induce us to alter the opinion there given. We do not think the differences pointed out by Dr. Bartlett between his typhoid and typhus sufficient to warrant his conclusion, that they are distinct forms, far less that they are the only forms of continued fever. Some of the points of difference referred to by him appear to us by no means made out, such as the distinction founded upon the different periods of life at which individuals are susceptible of the attacks of the two varieties. The absence of the Peyerian affection and diarrhea in our typhus does not appear to us to be so invariable as Dr. Bartlett represents it to be; nor is the distinction founded upon the darker and dustier hue of the eruption, or its extending to the extremities a sufficient reason for considering typhus a distinct form of fever.

With reference to the absence of lesion of the Peyerian glands, the dogma (for we must regard it as such) with which the author sets out, that there are only two forms of continued fever, has been the means of leading him into error; for he is evidently compelled to include under his description of typhus, forms of fever more distinct from typhus than it is from typhoid. He refers, for example, in illustration of his descriptions, to epidemics, in which, as we shall presently see, forms of fever prevailed very different from the common typhus of this country.

Nothing, we conceive, will tend more to give precision to our definition and arrangement of the different groups or forms of continued fever, than careful study and observation of the symptoms which characterize the great epidemics that occasionally pervade extensive districts. In such an epidemic, the features of a particular disease are displayed in all their different modifications and varieties, and it can then be seen what is essential to the species, what is distinctive, and what is to be attributed to various modifying influences.

In such an epidemic visitation, for example, *of scarlatina*, we might conceive that the essential characters of that disease were made out, its varieties distinguished, and a disease which had been long regarded as a distinct affection, under the name of malignant or putrid sore throat, received its proper place beside scarlatina simplex, and scarlatina anginosa.

Such, it may be anticipated, would be the result of a minute and extended observation of the forms of fever, as presented to us in the widely-spread epidemics of successive periods. An opportunity of this kind has occurred within the last twelve months, during which a very remarkable epidemic continued fever has pervaded the country, especially, the northern parts.

The works and papers quoted at the head of this article contain descriptions and notices of this fever as it appeared in Scotland, and a very cursory inspection of them will satisfy our readers that there are more forms of fever to be met with than those admitted by Professor Bartlett, and that the present form differs widely from the typhus described by him as the common fever of Great Britain. In the description of our typhus he has included certain points, such as the shorter duration of the attack, the benefits derived from bloodletting, and the occurrence of critical sweats, which are applicable in some measure to this epidemic fever, but not applicable at all to typhus. His description may justify his reference to the epidemics of former years, in which this fever undoubtedly prevailed, but goes far, at the same time, to prove that in order to comprise continued fevers under two species, he has been compelled to comprise more than one form under the name of typhus, his description of which appears to us to be taken from a mild form of continued fever, and an aggravated form of typhoid.

Before reviewing the peculiar type of fever described as the prevailing epidemic of the last ten or twelve months, we cannot but express our conviction that the study of typhus, as seen in different localities throughout this country, and in its different modifications in the same localities, during the prevalence of a great epidemic, would more than substantiate our general conclusion regarding typhoid and typhus, before referred to. Such a review of its modifications, we believe, would leave no doubt of the existence and *specific* character of a form of fever which presents certain general characters throughout all its modifications. Of these general characters, common alike to typhus and to typhoid, the most striking we believe are to be referred to the mode of invasion and propagation, the general appearance, the cutaneous eruption, the condition of the respiratory organs, the state of the circulation, the peculiar affection of the sensorial functions, and the progress of the malady.

Of the modifications which it presents, the only ones which appear at all material, are the greater frequency of abdominal organic lesions in some localities, as contrasted with the greater development of cutaneous eruption in others; varieties which afford no grounds, in our opinion, for referring affections, in other respects perfectly analogous, to distinct forms or species of continued fever. Remarkable varieties would be met with in such an epidemic, in the degree or intensity of the symptoms, in the severity of some cases as compared with others; but the general characters of the fever would still be sufficiently distinctive, both in kind and in duration, to establish the specific nature of the disease. The remarks of Dr. Bateman with reference to the London epidemic of 1810, as cited by Dr. Henderson, with his comments, are deserving of being repeated in this place, as bearing upon the point in question:

"Its character is greatly varied by the different circumstances in which it occurs, by the age, constitution, and previous health of the patient, by the intensity of the exciting causes, by the situation, and season, and by early neglect or mismanagement; but it is not more varied than other febrile diseases, the smallpox for instance, or scarlet fever, under similar circumstances, and examples of the most distinct modification which it undergoes are often observed in individuals of the same family. Thus, in the instance of a man and his wife, who were brought to the house of recovery together, the former was affected with the mildest symptoms of fever, which scarcely confined him to bed, and terminated in a speedy convalescence; while his wife was lying in a state of stupor, her skin covered with petechia and vibices; in a word, exhibiting the most fordmidable symptoms of the worst form of typhus. Yet these extreme degrees of the disease manifestly originated in the same cause; and it would be equally unphilosophical to account them different kinds of fever, and give them distinct generic appellations, as in cases of benign and confluent smallpox, which are generated in like manner from one contagion." (Bateman.)

"Every one," says Dr. Henderson, "who has seen much of typhus fever, must have met with many similar examples, proving the wide range which should be allowed, in the severity of the symptoms, to fever of that kind; and if degrees of severity of the symptoms were the only distinguishing features between the fever at present epidemic, and the less prevalent typhus, there can be no question that the attempt to distinguish them would be highly unphilosophical, because contrary to the analogy furnished by the examples of other diseases of whose identical nature in the extremes of mildness and severity there can be no doubt, and at variance with the testimony derived from the oneness of the source from which they have apparently sprung. But, by parity of reasoning, it would be highly unphilosophical to refuse to account those different kinds of fever, and to grant *them* different appellations, which differ widely, not in the degree, but in the nature of their symptoms, and which cannot be traced to one contagion, but in a very remarkable manner to two."

Diversities are observed in the nature of the symptoms developed in different cases of typhus, and in different epidemics, such diversities as have led, according to the prominence of one or another set of symptoms, to the denominations of gastric, pulmonic, cephalic, or bilious fevers, but these diversities do not affect the great features, and essential characters of the fever.

"Every disease," says Dr. Henderson, "has its accidents, no two cases of the same having features precisely alike, and it is, perhaps in a peculiar measure characteristic of typhus fever, that in different persons, at the same or at different periods of the year, and also in separate epidemics, local derangements may assume diverse forms, and may affect different viscera; while, notwithstanding, a residuary group of particulars remains common to all the cases, and serves to establish their identity as individuals of one peculiar disease."

The descriptions contained in the work of Dr. Cormack, in the different papers before us, of the epidemic which has recently prevailed, appear to us to justify the opinion advocated by Professor Henderson, that it is a different disease, a fever of a different and distinct *species* from our common typhus.

The earliest description of the peculiarities of this epidemic is contained in the brief notice published by Professor Alison, in the 'Scottish Medical Gazette.' While Dr. Alison apparently inclines to the idea that this kind of fever originated from the same poison as the usual typhoid fever of Edinburgh, he points out with great discrimination the characters by which it was distinguished from the strictly typhoid cases. The peculiarities referred to by him are: 1. The duration of the cases, which was uniformly short; the crisis occurring in most of them on the fifth or seventh day, very few being protracted beyond the ninth. 2. The absence of the measly eruption of typhus. 3. The frequent occurrence of jaundice, accompanied by more or less fulness and tenderness in the hypochondrium, and vomiting of green bile, or brownish matters like hare-soup. 4. The unusual degree of sickness and vomiting both in the jaundiced cases, and in others. 5. The constant, or almost constant, occurrence of a relapse, generally taking place on the fourteenth day. 6. The termination of the disease, in the great majority of cases, by profuse critical sweats. 7. The frequency of severe muscular pains of a rheumatic character, during and after the sweatings, and particularly after the relapse. 8. The mortality, which was very small, not exceeding one in thirty, in the cases which Dr. Alison had seen. In the notice given in the same journal by Dr. Arrott, of the epidemic as it was seen in the Dundee Infirmary, where it presented the same general features, with perhaps the more frequent occurrence of *black* vomiting, the mortality was singularly small, there being only seven deaths out of 672 cases. The last peculiarity noticed by Dr. Alison, is the fact that in every pregnant woman affected with the fever who came under his care, abortion took place.

In the paper of Professor Henderson, the differences between this epidemic and the common typhus constitute the principal object of his remarks; the distinguishing characters of the two fevers are, consequently, more fully pointed out, and contrasted. One of the most remarkable peculiarities, and the first which attracted the notice of Dr. Henderson, was the great frequency of the pulse in the new disease as compared with typhus, and the very different prognosis which was afforded by its frequency in the two diseases. This comparison, in consequence of the short duration of the epidemic fever, was necessarily limited to the first five days; and referring to examples in which it had been made, Dr. Henderson found the average frequency of the pulse in typhus on or before the fifth day, to be 100 per minute, while in the epidemic fever it was 123. He further observes, that according to common observation, a great frequency of the pulse in typhus fever, more particularly at an early stage, is deemed by practical men one of the most alarming symptoms next to those which proclaim immediate dissolution; while in this epidemic it appeared to have no special indication. In the epidemic fever, the pulse was frequently as high as 140 or 145 on the second or third days, and the crisis took place as usual on the fifth, without any indication of danger, while in typhus, even a less degree of frequency was followed by a very large proportion of deaths.

The next peculiarity noticed by Dr. Henderson is the mode of termination and rapidity of convalescence in the epidemic fever, as contrasted with typhus:

"In the typhus of former years," he observes, "and in exanthematic cases of fever, or unequivocal typhus of the last nine months, the decline of the disease has invariably, in my experience, been gradual from day to day, nearly a third of the whole duration of the febrile state having been usually consumed between the period when the fever began to amend and that of its total cessation. In the epidemic fever, on the contrary, a few hours witness commonly the transition from a state of extreme febrile heat, and frequency of the pulse to that of complete freedom from every trace of fever. In the ordinary course of things, we find the individual who at one visit presented a temperature of 104° or 106°, and a pulse of 130 or more, at the next, if the term of his fever has arrived in the interval, with a temperature of 98°, and pulse between 70 and 80. Nor is the critical discharge or secretion of the epidemic fever a less remarkable feature of difference between it and typhus. In a very few instances of typhus I have noticed a favorable change coincide with the occurrence of copious perspiration, but never a total cessation of the febrile state. In the great majority of instances, however, instead of copious perspiration coinciding, in typhus, with symptoms of amendment, it happens that it ushers in or accompanies a state of hopeless prostration, stupor, hurried breathing, and increased frequency of the pulse."

The constant occurrence of one or more relapses is next pointed out by Dr. Henderson as one of the distinguishing features of this fever. Using the term relapse in the strict sense of the term, as a return to or "repetition of the same malady through which the subject of it has recently passed," Dr. Henderson affirms that typhus never relapses. Out of 1600 or 2000 cases he himself never met with one instance of relapse, the only affections which might have been termed relapses being febrile attacks dependent upon the existence of some local inflammation occurring during the convalescence of the patient. Similar to his own experience is that of Dr. Perry, who states that of 1145 cases of typhus treated by him in the Glasgow Hospitals, in 1831, nineteen of the so-called relapses occurred, but all these were either cases of fever supervening upon some local inflammatory affection, and caught in the hospital, or local affections occurring during convalescence from the fever; and afterwards adds that "it is as absurd to talk of a relapse of typhus fever, as to talk of a relapse of smallpox or measles." The same evidence is afforded by Dr. Alexander P. Stewart in reference to the extensive epidemic of typhus which occurred in Glasgow: "I have never," he observes, "among thousands of cases, seen a single case of relapse, in the proper sense of the term, after the symptoms had begun to decline." (Edin. Medical and Surgical Journal, vol. liv, p. 300.)

In the fever under consideration, however, after the critical sweat of the fifth or seventh day, and a total though temporary remission, almost invariably a relapse, in the strict use of the word, that is to say a fresh attack of the fever, took place, generally on the fourteenth day; ushered in by shivering, and running nearly the same course as the primary attack. These relapses recurred not only once, but in a great many cases twice; and in a smaller number of cases a third, and even a fourth and fifth attack took place. With reference to the bearing of these upon the specific difference between typhus and the fever in question, Dr. Henderson remarks:

"That the difference in this respect between the two is a highly important one, I do not suppose that any one can doubt, and when I add that the cases of true typhus, which have occurred among the same class of the population, and at the same seasons, as the other disease, have presented no such tendency to relapse, or repetition of fever, as the latter does, it will appear, I conceive, that the liability to repetition cannot be esteemed other than a distinctive feature of the one disorder, and the absence of such a liability, a distinctive feature of the other, and that neither is dependent on accidental diversities in the epidemic constitution, either of the atmosphere or of the population, by which the effects of one and the same poison, might be presumed to be modified or altered."

The history of the epidemic, as relates to its progress, and propagation, and the persons affected by it and the common typhus, is next examined by Dr. Henderson, and some interesting facts are adduced which go to establish the non-identity of the two poisons from which the two fevers originate. Referring to the generally-acknowledged fact that an attack of typhus confers on the subject of it an immunity from a fresh attack for a considerable length of time, and to the circumstance, apparently established by the history of the present epidemic so far as it extends, that the same law pertains as regards it, Dr. Henderson cites a number of cases where the *two forms* of fever were exhibited in the same persons within a short period of time; some being affected with typhus soon after recovery from the epidemic, and some with the epidemic fever within a few weeks after their recovery from typhus. These facts, in the opinion of the writer, appear, and with justice, to lead to the conclusion not only that the dissimilarity of the fevers cannot be referred to the modifying influences of constitution and season, but that they must have arisen from poisonous influences originally different in their nature and source.

The history of the progress of the epidemic fever, and that of the cases of typhus occurring during the same period, develops other facts of interest in relation to the point in question; for, in the cases investigated by Dr. Henderson and others, the attack of the epidemic fever was invariably traced to intercourse with persons affected with the same form of fever, and in cases of typhus to persons affected with typhus, while in no instance could any case be referred to the contagious influence of the other form of fever. In some of the cases referred to, the two forms of fever prevailed in the same locality but in different houses; and in such instances the cases of the epidemic invariably came from that part of the locality where other cases of a similar kind prevailed, and those of typhus from the houses alone which the typhoid affection had visited,—facts which go very far to prove a difference in the kind of poison from which the two fevers originated.

Dr. Henderson does not dwell on the local affections presented so frequently in the epidemic fever, such as the jaundice, the affections of the urinary and biliary secretions, and of the blood, nor on the absence of the exanthematous spots of typhus, deeming these features to be less characteristic and peculiar; the former affections being sometimes observed in typhus, and the latter, the eruption, being occasionally absent to a greater or less extent in some of the epidemics of typhus.

In addition to the peculiarities pointed out by Professors Alison and Henderson, as characteristic of this epidemic, Dr. Cormack enumerates the sudden and violent invasion of the disease, and bronzing, leadening, or purpling of the countenance before and after seizure. With reference to the first particular, we believe that in a majority of cases of typhus there is a latent stage of some duration, and premonitory symptoms which indicate the approaching attack: nevertheless, we have, on the other hand, seen cases of typhus very suddenly formed without any previous indication of its approach; and on the other, we have observed many cases of this epidemic preceded by premonitory symptoms extending over one or two days. The peculiarity, therefore, we do not consider as characteristic of either affection, or at least, sufficiently well marked to establish a distinctive feature of the two affections. The latter peculiarity, the bronzing, leadening, and purpling of the countenance, in some instances described as deep and persistent, we do not remember to have remarked in the cases which have come under our own observation, nor can we refrain from expressing our surprise that a symptom, which Dr. Cormack mentions as one of the peculiarities which struck all the visitors to his ward as one of the most remarkable features of this epidemic, should have escaped the notice of all the other writers on the subject.

Dr. Cormack considers the peculiarity referred to as affording an interesting point of resemblance between the yellow fever of hot countries and this epidemic. (p. 86.) While it reminded him of the aspect of the inhabitants of the marshy districts of Italy, some of his visitors of the Walcheren epidemic, and others of the remittents and intermittents of Canada, the West Indies, and Italy, it coincides, he states, with the descriptions given by Audouard and others of the dingy, flushing, or leaden hue, which heralded the appearance of jaundice in cases of 'yellow fever.' While Dr. Cormack further rejects the idea of founding a diagnosis of fever upon the existence of yellowness or the occurrence of black vomiting, and shows that in some cases of typhus, in traumatic fever, in various epidemics, and in some kinds of poisoning, these symptoms are developed, he at the same time aims at establishing an analogy, if not identity, between this epidemic and the yellow fevers of tropical climates. This analogy he founds partly upon the resemblance in these features, and partly upon the pathology of the two diseases, giving at length a table of the appearances observed in his dissections, and a list of those observed in cases of yellow fever. With regard to the pathology of the two affections, we think there is little to found one, as we believe it must be acknowledged that the *true* pathology of either is still unknown. The post-mortem appearances observed by Dr. Cormack, which coincided with those of yellow fever, we would regard as not distinctive of either affection, for the same reason that Dr. Cormack considers the jaundice and black vomit as not distinctive of any fever, and the more so, as they are mostly those only which were connected with these particular symptoms. Of the other morbid appearances of yellow fever, Dr. Cormack contents himself with saying, "all these appearances are evidently the result of congestion; and many of them, not observed in my dissections, would, in all probability, have been observed had they been looked for." (p. 125.) On the whole, we think that the analogy drawn by Dr. Cormack between this epidemic and yellow fever is rather a hasty one, and somewhat fanciful. Although we would not fix upon the comparative mortality alone of the two affections as a *distinctive* character, modified as the termination of either may be by the difference of circumstances, we cannot avoid remarking the very great difference between them in this respect, and the discrepancy which exists between the results of Dr. Cormack's observations and those of the other observers as regards the mortality of the yellow cases with brown or black vomiting. The mortality in yellow fever is known to be very great, in some epidemics being as great as one in three, and in others even as high at the commencement of the epidemic as nineteen in twenty; and the black vomit is almost invariably regarded as a most unfavorable symptom. In the epidemic under consideration the mortality in Edinburgh is stated by Dr. Alison not to have exceeded one in thirty; according to Dr. Craigie, the mortality in his wards was not more than one in sixty; by Dr. Kilgour it is stated to have been less than one in thirty-five in Aberdeen; by Dr. Mackenzie, to have been about three and a half per cent. in Glasgow; and from Dr. Arrott's paper, it appears to have been only about one per cent. in the Dundee hospital.

The other circumstance, not less interesting, is the fact pointed out by Dr. Henderson, that there is no proof that the jaundice is of any consequence, or that it has produced a fatal event in a single instance. Although Dr. Cormack appears to regard the yellowness and black vomiting as occurring in the worst kind of cases, it is remarkable that in the epidemic as it prevailed in Dundee, where the mortality was so extremely small, and so much less than in Edinburgh, these symptoms were much more frequently observed and much more fully developed; and that the black vomit in particular, which was very rarely observed in Edinburgh, was very common there. Dr. Craigie altogether rejects the idea of the fever bearing any analogy to yellow fever. "It is scarcely possible," he says, "with any consistency in nosology, or common observation, to admit even the resemblance." (p. 416.)

Among the writers of the various papers before us, several conjectures are hazarded regarding the place which this fever should occupy in our nosology, besides those referred to in the preceding remarks. Rejecting the idea of its identity with yellow fever, or its origin from the poison of typhus, Dr. Craigie asks whether it is a remittent fever, a synochus, or identical with the *suette* or sweating-fever of Normandy; or whether it should be considered a gastric, a gastro-hepatic, a gastro-enteritic, or a rheumatic fever. These curious questions we are as little prepared to answer as Dr. Craigie himself appears to be, for he contents himself with propounding them. Into any question, indeed, regarding the nosology of fever we are disinclined to enter, believing as we do that nosology is fully as imperfect as it could well be; and that one and the same affection is very often described as illustrative, in part at least, of synochus, synocha, and typhus. But while we agree with some of the writers before us, that the descriptions given of *synocha* are, in some instances and to some extent applicable to a fever of this form, we are more concerned to trace with them its resemblance to the epidemics of former years, and to ascertain, if possible, whether a fever exists having specific characters, running a definite course, and periodically prevailing in an epidemic form, which may originate, it may be, from a definite cause or specific virus yet to be discovered. Inquiries directed to such an object, and extended to the epidemics of other diseases, may yet, it may be hoped, throw some light on the obscurity which invests the whole subject of the origin and cause of fevers.

In the article Continued Fever, by Professor Christison, published in the first volume of the 'Library of Medicine,'* the description of *synocha* is principally taken from two epidemics which prevailed in Edinburgh, the one in 1817-20, and the other in 1826-9, and leaves little doubt that those epidemics were identical with the one which has recently prevailed; or rather, that, along with the cases of typhus at that time epidemic, there prevailed also a species of fever, as at this time, steadily and uniformly distinguished from it as the *five-day fever*. In the mode of invasion, the state of the pulse, the duration, the occasional jaundice, the critical sweats, the relapses, the occurrence of rheumatic pains during convalescence, and other particulars, the cases were precisely alike. In

* Library of Medicine, vol. i, pp. 126-8, 140, 143-4.

the work of Dr. Welsh,* in which the former of these two epidemics is described, the resemblance between it and the recent one is still more fully illustrated. These two epidemics prevailed also in Ireland, along with typhus, and presented the same general characters. "There appears," says Dr. Mackenzie, (Med. Gaz., p. 27,) "considerable resemblance between the present fever and that described by Dr. Stoker, as prevailing along with typhus gravior in Dublin, in 1816. He speaks of it as a *typhus mitior*, its usual course being from three to nine days, generally terminating on or before the seventh day, but very apt to relapse on the third or fifth day, from the favorable change." We are indebted also to Dr. Mackenzie for the citation of Dr. O'Brien's description of the Dublin epidemic of 1826, which coincides so closely with that of the fever under consideration, as to merit repetition here :

"The other species of fever, or that of the new constitution, which constituted the bulk of this epidemic, was one of short periods, terminating in three, five, seven, or nine days; but the second of those periods was the most frequent. . . . . In this fever the chain of morbid actions was rapidly formed and rapidly terminated, and the disease developed itself with energy from the commencement. The access was sudden, and usually came on about mid-day. The person, previously in perfect health, would then be seized with sickness at stomach, headach, pain in the small of the back, and chilliness. On the approach of evening, all these symptoms increased, and the febrile paroxysm was fully formed; the chilliness increased to a rigor, and the nausea to vomiting, which harassed the patient for the first three or four days of his fever in the form of an empty straining, and frequently continued through its whole course. On the evening of the fifth or seventh day, the *exacerbatio critica* commenced, which, mostly with the intervention of a rigor, but very frequently without this symptom, terminated in a profuse perspiration, which continued through the night, so that on the following morning the crisis was complete, and we generally found the patient convalescent. We frequently received the glad tidings from himself, in the following words: 'Sir, I got the cool last night.' The *cool* however, was sufficiently visible in his countenance before he opened his lips; but, unfortunately, in many instances it proved only a delusive truce to his sufferings. The patient was destined, perhaps, to be harassed by one, two, or three relapses, which prolonged the whole duration of his illness even beyond that of the most protracted typhus. In fact, the liability to frequent relapses was one of the most striking characteristics by which this fever was distinguished from all previous epidemics—at least, which happened in our time. . . . . . . Relapses, generally speaking, were milder and shorter than the original fever; but to this many exceptions occurred. The general symptoms of the summer variety of this fever, in addition to those already mentioned, were—acute headach; delirium, always active, sometimes phrenitic; rapid and hard pulse; white tongue, with florid edges, but sometimes natural; muscular and arthritic rather than deep-seated pains, or, as they were termed, 'pains of the bones,' not accompanied, however, by swelling of the joints except in a few instances; the skin, in many cases, of a light yellow tinge, and sometimes, though rarely, assuming the intense icteroid yellow, characteristic of jaundice and true yellow fever."†

A further proof of the identity of the two epidemics is derived by Dr. Mackenzie from the fact, that the same affection of the eye followed many of the cases of 1826 as that which is described by him in his interesting account of the post-febrile ophthalmitis of the recent epidemic in Glasgow.

* On the Efficacy of Bloodletting in the present Epidemic Fever of Edinburgh. By BENJAMIN WELSH, M.D. 1819.

† Transactions of the Association of Fellows and Licentiates of the King and Queen's College of Physicians in Ireland, vol. v, pp. 266-512; Dublin, 1828.

The prevalence of epidemics of the same kind at earlier periods in Ireland, appears probable from the statements of Dr. Rutty in his 'History of the Diseases of Dublin during forty years,' also cited by Dr. Mackenzie. "In July, August, September, and October, 1739, a fever prevailed, which was attended with an intense pain in the head. It terminated," continues Dr. Rutty, "sometimes in four, for the most part in five or six days, sometimes in nine, and commonly in a critical sweat: it was far from being mortal. I was assured of seventy of the poorer sort at the same time in this fever, abandoned to the use of whey and God's good providence, who all recovered. The crisis, however, was very imperfect, for they were subject to relapses, even sometimes to the third time."* He describes the same remittent fever as occurring also in 1740, 1745, 1764, and 1765.

The limits of this article prevent us from entering further into the history of this form of fever than it has been brought casually before us in the papers under review. These remarks and the references to Dr. Rutty's work given below may suggest the mode in which the inquiry might be extended.

Leaving the history and nature of this epidemic fever with these general remarks, one or two facts of considerable interest in relation to the pathology of the affection are recorded in the works before us, which demand attention.

The pathological appearances mentioned by Dr. Cormack are referred principally to congestion of the internal organs, and are described by him as consisting mostly in a dark-coloured state of the gastro-intestinal mucous membrane, with occasional extravasation of patches of blood beneath it. With regard to the state of the blood, his remarks are as follows:

"That the blood really is in a dissolved state was made perfectly manifest to us—*first*, by the imperfect coagulation which it underwent when drawn from the veins of patients, a homogeneous spongy mass being formed in place of a firm fibrinous clot, with a supernatant serosity; *second*, by the ecchymosis which was uniformly observed to surround flea-bites or other slight injuries of the skin; *third*, the frequent occurrence of purpurous spots; *fourth*, the hemorrhages; and *fifth*, the discoveries made by the microscope. Professor Allen Thomson had the goodness to lend me his able assistance in examining the blood of a number of my patients by means of the microscope. A few drops were taken from the thumbs on the same day (Oct. 24th) of about a dozen persons, some of them in pyrexial and others in the apyrexial state of the disorder; and it was found that in all of them there was an unusual number of pus-globules; (?) and in some cases, in addition to this, all the globules were found serrated and notched." (pp. 113-4.)

In a considerable proportion of the cases treated by Dr. Henderson, the spleen was discovered to be affected; in some instances it was enlarged so much as to be easily distinguished projecting beyond the lower and anterior margin of the hypochondrium, and in others the enlargement was discovered by percussion only. This enlargement was accompanied with tenderness over the spleen, and pain in the region of that organ—a symptom complained of by many in whom enlargement was not determined. The enlargement of the organ yielded readily to local remedies; it was in some instances accompanied by symptomatic fever, which

* Chronological History of the Weather and Seasons, and of the prevailing Diseases in Dublin. By JOHN RUTTY, M.D.; London, 1770, p. 75, pp. 90, 130, 303-4, and 319.

ushered it in, or continued during the remission from the paroxysm of the epidemic.

One of the most interesting facts connected with the pathology of the recent epidemic was the discovery of urea in the blood and serous fluid of the ventricles of the brain, in some of the patients affected with symptoms of cerebral derangement. The suspicion of this morbid condition was suggested to Dr. Henderson partly by the occurrence of convulsions in several cases in which there was no jaundice, and partly by a case in which symptoms of oppression and confusion of mind, accompanied with diminution of urine, was relieved by the occurrence of diuresis after the exhibition of diuretics. In two cases which subsequently occurred, exhibiting indications of cerebral oppression, the state of the urine was attended to and the blood analysed. In both cases the symptoms came on after the critical sweat; and the urine was somewhat diminished in quantity, although not materially. In one case, the patient, after oppression, stupor, and a repetition of convulsive fits, died, and three drachms of serum from the ventricles of the brain, with some clots of blood from the head, were examined. "Crystals of nitrate of urea were obtained in moderate abundance from the serum of the brain, and a very considerable quantity from the blood." In the other case, after somnolence, confusion, and languor, blood drawn from the arm yielded crystals of urea in small quantity. The character of the symptoms which succeed *suppression of urine*, and the explanation afforded of such cases by the detection of urea in the blood, have been long known to the profession. The possibility that a similar condition of the blood might be found to occur in other affections, and might afford the true explanation of sudden death in diseases not expected to terminate thus, was suggested, we believe, by Dr. Christison. But we have here, for the first time, met with the realization of this conjecture, in the proof afforded by the observations of Dr. Henderson, that such an event may prove the immediate cause of death in cases of *fever;* and that, too, even in cases where the obvious cause which might give rise to the presence of urea in the blood—namely, the *suppression* of urine—was absent.

The facts elicited in these observations led to the further investigation of the subject in Dr. Henderson's wards, by Mr. M.W. Taylor; and in other two cases of fever—one of the epidemic, and one of typhus, both exhibiting the development of cerebral symptoms, with *diminution* of the urinary secretion—a circumstance otherwise indicating a favorable prognosis—urea was detected in the blood. The existence of urea in the blood, says Mr. Taylor (Scottish Med. Gaz., p. 289), in other cases "has been inferred from the occurrence of those symptoms of disorders of the nervous centres, which we know to be the consequence of its undue accumulation in the circulation. These phenomena have been observed in those cases in which, from some cause or other, the daily discharge of urine has undergone material diminution. This appears to take place chiefly at that critical period of the fever marked by copious sweating, at which the febrile symptoms begin to subside, or during the apyretic intervals between the attacks. Professor Henderson was the first who drew the attention of the profession to the fact of the occasional presence of urea in the blood, at this stage of the fever, under the above-mentioned circumstances." The inferences deduced by Dr. Henderson from these observations are of great practical importance, and may prove to be so in relation to the treatment of other fevers besides the one under consideration :

"I have adverted," he observes, "to the jaundice which is apt to occur in the course of the fever, and was once inclined to regard it as a very formidable symptom. I am in a condition to assert now, however, that there is no proof that the jaundice is of any consequence,—no proof that the jaundice has caused those symptoms of cerebral disorder which have been ascribed to it, or has produced a fatal event in a single instance. One of the cases which I have detailed was a case of jaundice; and if the blood had not been examined, the death would have been ascribed, in that instance, as it has been in not a few others, to the jaundice. Henceforth, before any one can assert that he has lost a case of this fever from jaundice, he must be prepared to show that there was no urea in the blood. The examples of complete jaundice which have come under my care have amounted only to six. Five of them had not a single symptom which made them differ from the ordinary cases, excepting the yellowness, and that was as deep as in the fatal case, in which the jaundice and the accumulation of urea occurred together. It will, therefore, be no small advantage if the facts which I have been able to determine respecting the latter, shall teach us to inquire carefully into the state of the urine at that period of the disease during which the vigilance of the physician has usually become relaxed, from the supposition that it was no longer necessary, and more especially in such cases of jaundice as present symptoms of cerebral disorder; for in these circumstances it appears that our calomel, our cupping, leeching, and blistering the right hypochondrium should give place to the remedies which act upon the kidneys."

To what extent those principles may be applicable in the treatment of typhus and other fevers, remains yet to be determined ; but many of the facts before us would tend to show they were peculiarly applicable in the treatment of the more formidable cases of this fever. Dr. Smith, in his interesting and valuable paper on the Epidemic as it appeared in Glasgow, states that, in the majority of cases where there was great depression of the powers of life, the urine was considerably suppressed, and, in old people particularly, the patient was apt to become lethargic or comatose. (p. 69.) His experience extended to 1000 cases, occurring in the lowest, filthiest, and most densely-populated part of the city. Of that number 43 died, and he states that, "when death occurred the patients seemed to sink into a state of lethargy or coma, from which they could be easily aroused, and would answer questions put to them with considerable correctness ; and in this condition I have seen them remain for a couple of days." (p. 73.) These symptoms, it may be supposed, were probably dependent upon the cause pointed out by Dr. Henderson, and might have been benefited by treatment directed to the elimination of urea from the system.

Dr. Smith's paper contains some valuable facts of a statistical kind, in relation to this fever. Jaundice, which he believes did not interfere with the progress of the disease, occurred in 384 cases, petechiæ in 314, diarrhea or dysentery in 167, bronchitis in 132, and relapses in 112 cases. Pneumonia and pleuritis were only seen accompanying the relapse, and were present in 3 cases each. Cynanche laryngea occurred in 9 cases; epistaxis in 13. Of the 43 deaths, diarrhea was present in 10, jaundice in 9, vomiting in 4, diarrhea, jaundice, petechia, &c. in 12, dysentery in 2, and pneumonia in 1.

The last, and not the least interesting of the facts connected with the recent epidemic, was the occurrence, in a considerable number of cases, of a peculiar inflammatory affection of the eye, taking place during or after convalescence. The affection is described by Dr. Mackenzie under the name of *post-febrile ophthalmitis*. It appears to have occurred in greater proportion in Glasgow than in the cases treated in Edinburgh and elsewhere. In the Glasgow Eye Infirmary 36 cases occurred between the 8th of August and the 31st of October. It was characterized by amaurosis, or imperfect vision, affecting one or both eyes, and inflammatory action, extending from the retina to the other tunics of the eyeball. The symptoms are thus described by Dr. Mackenzie :

"The characters of the disease appear to be, in the first instance, that of congestion, followed by inflammation of the internal parts of the eye, and especially of the retina, producing great imperfection of sight. This is succeeded by evident inflammation of the iris and sclerotica, the disease extends to the capsule of the lens and sometimes to the lining membrane of the cornea; there can be little doubt but that the choroid is also inflamed; while the conjunctiva remains in general but slightly affected. The part which the sclerotica takes in the disease is plain enough, from the intense injection of the blood-vessels which lie on its surface, and which, derived from the muscular and anterior ciliary arteries, are seen running in radii towards the cornea. The change of colour in the iris, the contracted state of the pupil, and the tags of adhesion between the edge of the pupil and the capsule of the lens, show the part which the iris takes in the disease. The internal membrane of the cornea, and the anterior crystalline capsule, especially the latter, are extremely muddy, showing their participation in the inflammation. The whole walls of the aqueous cell seem, in some cases, as if coated with a thin layer of lymph, of a yellowish green colour. The great deficiency of sight is not explicable from the mere muddiness of these parts, and is, besides, often the earliest symptom of the disease, showing an affection of the retina. At an early period the pupil is sometimes dilated, and does not become contracted till the inflammation embraces the iris. If not promptly combated by the appropriate remedies, the cornea and sclerotica become preternaturally flexible under the pressure of the finger, showing that the disease has extended to the vitreous body. In one case I found the cornea very flexible in the amaurotic stage, before there was any appearance of inflammation. The lacrymation is very considerable, and seems to be connected, not so much with the state of the conjunctiva, as with the pain in the interior of the eyeball. The severe pain in and round the eye, aggravated during the night, is exactly similar to what attends rheumatic and syphilitic ophthalmia, and may partly be ascribed to the pressure exercised upon the ciliary nerves within the eye by the inflamed parts, partly regarded as a direct neuralgic affection, such as we often meet with in the six branches of the fifth nerve which emerge from the orbit, when there is no evident inflammation. It is, in general, only after the iris and sclerotica have taken part in the disease that the patient complains of the ocular and circumorbital pain. So long as the disease is confined to the retina there is little or no pain. Hence, the patient is less alarmed than he should be by the mere dimness of sight, which, indeed, from only one eye being generally affected, may scarcely attract his attention. Even *photopsia*, in the early stage is not much complained of; in the last stage, *muscæ volitantes* form a constant symptom. Although, in by far the greater number of cases, all the textures of the eye suffer in this disease—on which account it may be designated an *ophthalmitis*—it sometimes happens that the inflammation is confined to one or two textures only."

These attacks occurred at various periods from three to sixteen weeks after the accession of the fever. In several instances it occurred about two weeks after convalescence from the relapse, but generally later.

The same disease, it would appear, occurred after the Dublin epidemic of 1826, and was described by Mr. Hewson, Dr. Reid, Dr. Jacob, and Mr. Wallace, confirming the views we have adopted of the identity of the epidemics.

The treatment pursued with the greatest amount of success by Dr.

Mackenzie was similar to that followed in cases of rheumatic and syphilitic iritis, and consisted principally of bloodletting, followed by calomel and opium, dilatation of the pupil with belladonna, and counter-irritation.

This affection appears to have been much more frequent in Glasgow than elsewhere, as it is not noticed by the other writers, with the exception of Dr. Cormack, who refers to only three cases, as having been seen by him. This apparent difference, however, may depend upon the circumstance of its occurring so long after the fever that, in most instances, the patients must have been dismissed from hospital previous to its development, and would probably apply to some dispensary for advice when the eye became affected. Dr. Cormack has transferred the whole of Dr. Mackenzie's interesting paper on the ophthalmitis to his pages, where it supplies what would otherwise have been a great desideratum. The rest of Dr. Cormack's work consists, in a great measure, of the reports of cases occurring in the wards of the Fever Hospital under his care, and although it bears some marks of haste, Dr. Cormack has rendered a service to the profession in recording at some length the features which struck him as distinguishing this fever.

The Treatment of this epidemic appears to have been simple, and to have been directed mostly to the mitigation of sufferings, or to the subduing in some instances of local complications. Any attempts made to cut short the fever, or to accelerate the crisis, or even to prevent the relapses, seem to have failed. The disease, apparently, ran its course, and terminated at the usual period, and in the usual manner, by profuse sweating, whatever mode of treatment was adopted, equally as in those cases where the patients did not enjoy the benefit of medical treatment at all. Such cases were numerous; for, from the great prevalence of the epidemic, the hospitals, although in some instances greatly enlarged, were quite inadequate for the accommodation of all the applicants, and the dispensary physicians and pupils were equally unable to attend all the patients who sent for assistance at their own homes.

Dr. Smith appears to have directed his attention particularly to the possibility of preventing the fever from running through its usual course. His evidence on this subject is interesting, and exactly such as we should have expected:

"On the first appearance of the present epidemic amongst us, I embraced the opportunity of testing extensively the effects produced by emetics, diaphoretics, and other evacuants, in cutting short the progress of the disease. In upwards of 150 cases seen on or previous to the third day of attack, this mode of treatment was adopted; and, in addition, about fifty patients labouring under the symptoms of threatened relapse, were subjected to the same treatment as those in the incipient stage; but in all I have never seen a satisfactory arrestment of the fever: on the contrary, I have been led to believe, from what has fallen under my own observation, that this disease cannot be cut short by any means." (Op. cit., p. 73.)

The treatment adopted in the management of this fever is but briefly stated, and in some instances only incidentally alluded to, by the writers before us, with the exception of Dr. Cormack, who necessarily enters into the consideration of it at some length. Following his remarks, we shall refer to the opinions of the others as they happen to relate to the different points at issue.

Assuming the impossibility of cutting short the disease, Dr. Cormack sets out with the grand and indisputable generalization, that the great object, in reference to the prevailing epidemic, was "*to obviate the tendency to death.*" This fortunately was not great, and the physician, consequently, had the less to do. The states considered by Dr. Cormack as most apt to cause death, and therefore "to be anxiously looked for, and if possible, promptly corrected," are

"1st. Congestion of the mucous membrane of the stomach and intestines, terminating in effusion of blood and subsequent destruction of large portions of this tissue.

"2d. Congestion of one or more of the abdominal viscera, particularly of the liver and kidneys, disabling them from the performance of their secretive functions, thereby causing bodies to circulate with the blood, which ought to be separated from it, and which bodies we know act as poisons when not so eliminated from, or when directly introduced into the circulation.

"3d. Debility and sinking." (p. 150.)

With regard to the first state, in two or three of the cases recorded by Dr. Cormack, debility and sinking, followed by death, appear to have been occasioned by hemorrhage from the stomach or bowels; and in those cases, exudation of dark-coloured blood, on and under the mucous membrane of the stomach and intestines, was found to a considerable extent upon examination after death.

As to the second cause of death, it refers principally to the development of urea in the blood, as pointed out by Dr. Henderson; but we have already sufficiently commented upon that point, and on the important relations which it bears to the treatment of this and, probably, of other fevers. In as far as the cause referred to (congestion of the abdominal viscera) is stated to have operated through the medium of the liver, we think there is every reason to believe, from the evidence before us, that the cases affected with jaundice were not more fatal than the cases not so affected.

The debility and tendency to sinking were produced, we think, most commonly by the occurrence of diarrhea, or by the profuse sweating which formed the most frequent crisis of the fever.

Bloodletting, both general and local, appears to have afforded great relief, in the experience of Dr. Alison and others, to the violent headach, and other uneasy feelings of the early stage of the fever; and could be had recourse to without those apprehensions of protracted weakness and exhaustion, which form the great obstacle to contend with in most cases of typhus fever. The tenderness in the epigastrium or hypochondriac regions, with indications of enlargement of the spleen, appear to have yielded readily to the application of leeches. Dr. Cormack rather inclines to discountenance bloodletting, convinced that the latter symptoms give way with equal readiness after the diligent use of warm fomentations, and the former after the use of purgatives and cold applications to the head. He thinks that the headachs return soon after the bloodletting, although temporarily relieved, and appears to dread the debility which may ensue upon the use of such a remedy.

Diaphoresis appears to have occurred spontaneously on the critical day, and to have been as profuse and as beneficial when no diaphoretics were used as when they were; and although they were pretty constantly employed, they did not appear, in any instance, to accelerate the crisis, but they afforded relief or satisfaction to the feelings of the patient.

The administration of opium, and the external application of blisters or sinapisms to the epigastrium, were found very serviceable in allaying one of the most troublesome symptoms—the vomiting. In some instances, Dr. Cormack tried creosote, in others hydrocyanic acid, for this symptom, and with marked success.

The application of cold to the head for the purpose of allaying headach, and the use of cold or of hot or tepid sponging of the surface of the body in allaying restlessness, were found by Dr. Cormack to be very useful and grateful to the patients. His remarks on the application of heat and moisture to the skin are worthy of being quoted:

"Although of opinion, that all diaphoretic and sudorific drugs are of little advantage, and that violent sudorific doses are injurious, I am yet of opinion that I have very often seen the best possible results from other means employed for the purpose of diaphoresis and sweating, such as the wet blanket, the partial warm bath, and tepid sponging. The general warm bath is apt to produce exhaustion. I had three young patients affected with ardent fever and dry hacking cough, closely wrapped up in a blanket wrung out of hot water. Above this were placed several dry blankets. They sweated most copiously from ten to fourteen hours, and were then removed to a dry bed, where they all sweated again so freely as to require to have their linen changed. They emerged from this sudorific regimen, perfectly free from fever, cough, and pains, but excessively weak and languid. A child of four years old, when in the initial rigors of the relapse, was treated in the same way with equal benefit. I was led to try this plan from having recollected that Dalrymple, in the fever of Carthagena in 1740, and Jackson subsequently, in the yellow fever of the West Indies, had found that decided and immediate benefit had resulted from rolling the patients in blankets wrung out of hot salt and water. Had the supply of bedding been adequate, this method would have been in general use." (pp. 157-8.)

It is to be regretted that Dr. Cormack does not mention the day of the fever on which this hydropathic treatment was used, in the three young patients subjected to it, and that the remedy was not more extensively tried by him.

The administration of purgatives was found useful in mitigating the symptoms, and particularly in relieving the headaches. Croton oil was prized by Dr. Cormack, because, in spite of its activity, he did not find that it produced irritation; but that, on the contrary, it appeared to soothe the gastric irritability and nervous excitement, and to leave the patients little exhausted, even after its free and frequent action.

Mercurial purges appear to have been preferred by Dr. Craigie and others in the treatment of the jaundiced cases, but, after trying them extensively, Dr. Cormack concludes that the cases went on at least as well without them.

The rheumatic pains with which the patients were affected during convalescence, do not appear to have been very amenable to treatment. From the use of colchicum, Dr. Cormack does not think his patients experienced any benefit.

Astringents, chalk mixtures, acetate of lead and opium, acetate of lead and squills, &c., were found very serviceable in the cases complicated with diarrhea, dysentery, and bronchitis; and tonics, with the occasional administration of stimulants, in the debility which, in many cases, accompanied and followed the crisis.

The chlorate of soda was used, but without any decided evidence of its advantages.

The sulphate of quinine, both in small and large doses, frequently repeated, and other reputed anti-periodic remedies, were tried in a great many cases, with a view to prevent the occurrence of relapse; but, although Dr. Cormack conjectures that the former remedy modified or delayed the relapse, none of them appeared to have any effect in the prevention of its occurrence.

None of the writers of the various notices under review had any doubts regarding the contagious nature of this fever, and numerous facts are recorded by them, both in proof of this fact and of the different sources from which the cases affected with it, and those affected with typhus, originated. To these we have already alluded; but we cannot conclude these remarks, without referring to the evidence of Mr. Ramsay, inspector of lighting and cleaning in Edinburgh, as to the influence of lime-washings of the dwellings of the poor in arresting the progress of this epidemic. This is contained in a very interesting Report, prepared by Mr. Ramsay for the Commissioners of Police, on the means of promoting the health and cleanliness of the lower classes at the public expense. It contains some interesting statements regarding the cleansing and whitewashing of those localities where fever prevailed.

The number of cases of fever compiled from the books of the Royal Infirmary and Fever Board alone, as coming under the notice of those institutions during a period of eleven months and two days, was as follows:

| | | | |
|---|---|---|---|
| In January, there were | | 48 | cases. |
| February | ,, | 75 | ,, |
| March | ,, | 77 | ,, |
| April | ,, | 91 | ,, |
| May | ,, | 114 | ,, |
| June | ,, | 161 | ,, |
| July | ,, | 267 | ,, |
| August | ,, | 376 | ,, |
| September | ,, | 636 | ,, |
| October | ,, | 489 | ,, |
| November | ,, (including 2d December) | 674 | ,, |

Making a total number of 3008 cases.

of which 566 only occurred during the first six months of the year, and 2442 during the last five months and two days.

The business of whitewashing the fever localities was commenced under Mr. Ramsay's active superintendence, on the 14th of September, and continued to the 30th of October, and less extensively up to the 14th of December, during which period, he states there were washed

"690 staircases,
1531 passages,
2120 apartments, exclusive of
1212 places cleaned previous to that date.

Making a total of 5553 different places, at a total expense of £77."

And the result of Mr. Ramsay's observations, in reference to the effect of those cleanings on the progress of the fever, was as follows:

"The effect of these lime-washings on the epidemic has been matter of great interest to myself; and I have watched with the most anxious care to ascertain whether any new cases of fever occurred in dwellings previously subjected to purification; and I have pleasure in saying, that out of a great number of cases reported to me, with two exceptions, the whole turned out to be cases of relapse. That other cases must have occurred, there can be but little doubt; but notwithstanding of the pains I have taken to discover them, I have hitherto failed in doing so."

In concluding these remarks we cannot refrain from expressing our conviction that contributions similar to those which we have in the papers under review, describing the history and progress of this epidemic, as it has appeared in different parts of the empire, for we believe that it has traversed the island, would afford a series of facts of great value to medical science; and that a collected view and digest of the facts thus obtained might throw considerable light on the subject of fever. By a historical investigation we believe it might be shown that notices of such a fever as that by which we have been recently visited, could be traced back as far as the times of Hippocrates. That such an investigation, or such a general view of the history and progress of this and former epidemics of the same fever, would, at the present time, develop the source of those poisonous influences by which it or other fevers may be generated, we are not sanguine enough to anticipate; but we cannot doubt that it would contribute to give precision to our present vague and unsatisfactory views regarding the forms and varieties of continued and remittent fevers, and put us one step in advance in those investigations into their causes which it is most desirable for us to effect.

---

